# A cost‐effectiveness analysis of lusutrombopag for thrombocytopenia in patients with chronic liver disease in Japan

**DOI:** 10.1002/jgh3.12597

**Published:** 2021-06-26

**Authors:** Norikane Miki, Sachie Inoue, Hidetoshi Shibahara, Kenji Kurazono, Rodolphe Perard, Ryosuke Tateishi

**Affiliations:** ^1^ Shionogi & Co Ltd. Osaka Japan; ^2^ Crecon Medical Assessment Inc. Tokyo Japan; ^3^ Shionogi B. V. London UK; ^4^ The University of Tokyo Tokyo Japan

**Keywords:** chronic liver disease, cost‐effectiveness analysis, lusutrombopag, thrombocytopenia, thrombopoietin receptor agonist

## Abstract

**Background and Aim:**

Thrombocytopenia is a frequent hematological condition in chronic liver disease (CLD) patients increasing the risk of bleeding in patients undergoing invasive procedures. Without an alternative, clinical guidelines recommended the use of platelet transfusion (PT) prior to procedure to prevent this bleeding risk. Lusutrombopag (LUSU), an orally active, small‐molecule thrombopoietin receptor agonist, was developed as an alternative to PT. The objective of this study was to evaluate a cost‐effectiveness of LUSU as a potential alternative to PT in Japan.

**Methods:**

A cost‐effectiveness analysis of LUSU relative to PT was conducted by a simulation model consisting of a decision tree combined to Markov model. Quality‐adjusted life years (QALYs) were used as an indicator of efficacy, and the analysis was conducted from the Japanese public healthcare payer's perspective. The time horizon of the analysis was 50 years (a lifetime) and the discount rate was set at 2%.

**Results:**

LUSU gained 6.1803 QALYs with an expected lifetime costs of 2 380 219 JPY compared to PT with 6.1712 QALYs gained and expected lifetime costs of 2 382 908 JPY. Thus, LUSU was deemed dominant compared with PT. Based on probabilistic analyses, the chance of LUSU being dominant and the incremental cost‐effectiveness ratio being below 5 million JPY/QALY was estimated at 51.8% and 78.3%, respectively, demonstrating the robustness of the results.

**Conclusions:**

LUSU was evaluated as an efficacious and cost‐saving treatment option for Japanese CLD patients with thrombocytopenia who required a planned invasive procedure compared with PT and economically should be considered as an alternative treatment.

## Graphical abstract

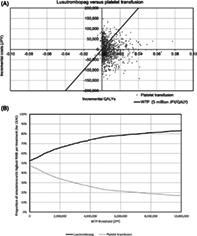

Thrombocytopenia is a frequent hematological condition in chronic liver disease (CLD) patients increasing the risk of bleeding in patients undergoing invasive procedures, and the use of platelet transfusion (PT) was recommended prior to procedure to prevent this bleeding risk. This study evaluated the cost‐effectiveness of lusutrombopag (LUSU) as a potential alternative to PT in Japan by using a simulation model consisting of a decision tree combined to Markov model. Compared with PT, LUSU was found to be an efficacious and cost‐saving treatment option for Japanese CLD patients with thrombocytopenia who required a planned invasive procedure.

## Introduction

Chronic liver disease (CLD), which includes several etiologies, such as viral hepatitis and alcoholic liver disease, is generally characterized by gradual, irreversible liver damage and multiple comorbid complications. Thrombocytopenia is often defined as a platelet count <150 × 10^9^/L.^1^ Regardless of CLD etiology, thrombocytopenia and CLD are often comorbid, with thrombocytopenia developing in 64–84% of CLD patients.^1^, ^2^ Furthermore, severe thrombocytopenia, defined as a platelet count <50 × 10^9^/L occurs in 1–2.6% of the CLD population.^1^, ^3^, ^4^ Severe thrombocytopenia is associated with an increased risk of potentially serious bleeding events during or after an invasive procedure.^5^ Severe thrombocytopenia in CLD may delay or prevent the diagnostic and therapeutic procedures critical to the care of this patient population, potentially exacerbating the condition of a patient or increasing the patient's morbidity and mortality.^2^, ^6^ Currently, guidelines for the use of platelet transfusion (PT) concentrates based on scientific evidence published by the Japanese Ministry of Health, Labour and Welfare^7^ recommends the use of PT to perform invasive examinations or procedures to prevent hemorrhage in CLD patients with severe thrombocytopenia. On the other hand, clinical practice guidelines for liver cirrhosis 2020^8^ recommend the use of thrombopoietin receptor agonists for cirrhosis patients with thrombocytopenia who required a planned invasive procedure.

Lusutrombopag (LUSU) is an orally active, small‐molecule thrombopoietin receptor agonist licensed for “improvement of thrombocytopenia associated with CLD in patients undergoing an elective invasive procedure,” launched in December 2015 in Japan to treat thrombocytopenia prior to a planned procedure. LUSU at 3 mg is administered orally once daily for 7 days prior to the planned invasive procedure.

UK's National Institute for Health and Care Excellence (NICE) deemed that LUSU is likely to save money for the National Health Service (NHS); NICE, therefore, recommends the use of LUSU as an option for treating severe thrombocytopenia in adults with CLD undergoing planned invasive procedures.^9^

The Japanese society has been facing marked changes with the aging population and soaring national medical care expenditure, hence the increased interest in the budget impact and cost‐effectiveness of newly introduced medical technologies. The full‐scale cost‐effectiveness evaluations were introduced for price adjustments in Japan from 2019.^10^ The objective of this analysis was to evaluate the cost‐effectiveness of LUSU compared with PT in Japan.

## Methods

### Overview

The cost‐effectiveness analysis (CEA) of LUSU relative to PT was conducted by a simulation model consisting of a decision tree combined to Markov model. The model considers the efficacy of LUSU as measured by two randomized clinical trials (RCTs) targeting Japanese CLD patients with severe thrombocytopenia who required a planned invasive procedure (L‐PLUS1 and P2b). Quality‐adjusted life years (QALYs) were used as an indicator of efficacy, and the analysis was conducted from the Japanese public healthcare payer's perspective and included only direct medical costs. The time horizon of the analysis was set at 50 years (a lifetime). A 2% discount rate was used to discount QALYs and costs according to the guideline of cost‐effectiveness evaluation in Japan published by Ministry of Health, Labour and Welfare (MHLW).^11^

### Model structure

**Figure 1:**
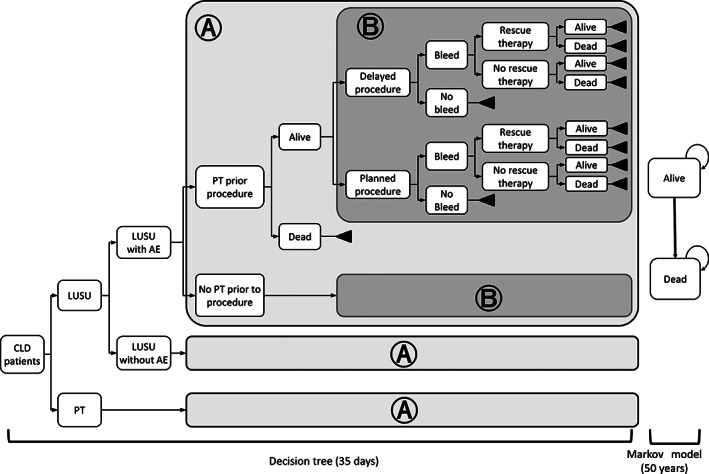
Model structure.AE, adverse event; CLD, chronic liver disease; LUSU, lusutrombopag; PT, platelet transfusion.

The cost‐effectiveness model incorporated a decision tree model during the period receiving an invasive procedure (a 35‐day time horizon), and a Markov model evaluating dead and alive thereafter. The structure of the model is shown in Figure 1. The initial decision tree model included the following events: receiving PT prior to the planned invasive procedure, delay of the planned invasive procedure, bleeding following planned or rescheduled invasive procedure, receiving rescue therapy following bleeding, and death from bleeding. PT was assumed to be the rescue therapy following bleeding. As an adverse event (AE) of LUSU, the development of thrombosis was considered. Mortality data of each Child–Pugh category were referred to the report of Shindo et al.^12^ and adjusted by the proportion of severity defined with the Child–Pugh category at baseline to estimate the mortality of CLD patients.

### Additional benefit

To evaluate the additional benefit of LUSU among Japanese patients, this study used the results of a meta‐analysis of L‐PLUS1^13^ and P2b^14^ clinical trials, which were Japanese, multicenter, randomized, double‐blind, parallel‐group, placebo‐controlled Phase 3 and Phase 2b studies, respectively, targeting patients with CLD (Child–Pugh class A and B) and platelet count of <50 × 10^9^/L at screening. The primary efficacy endpoint for L‐PLUS1 was the proportion of patients who required no PT prior to the primary invasive procedure. The trial achieved the primary endpoint, with significantly more patients who received LUSU requiring no preoperative PT (LUSU, 79.2%; placebo, 12.5%). The P2b study reported results consistent with those of L‐PLUS1.

A meta‐analysis of the L‐PLUS1 and P2b studies was conducted regarding the risks of patients receiving PT prior to the planned invasive procedure and bleeding following a planned or rescheduled invasive procedure. The baseline characteristics and the number of events or target patients used in the analysis are shown in Table S1, Supporting information.

A meta‐analysis of the LUSU trials was conducted using R statistical software (version 3.6.2), and the random effect model was adopted to consider the heterogeneity. Although the risks of delay of the planned invasive procedure and receiving rescue therapy following bleeding were also outcomes assessed in the clinical trials, the reliability of the estimated values was considered poor because there was only one event in both groups. Therefore, meta‐analyses for these parameters were not performed.

Based on the results of the meta‐analysis, the odds ratio (OR) of the risk of patients receiving PT prior to the planned invasive procedure and bleeding following a planned or rescheduled invasive procedure in the LUSU group compared with the placebo group was 0.04 (95% confidence interval [CI]; 0.02, 0.11) and 0.44 (0.19, 1.02), respectively (Figs. S1 and S2).

### Model inputs

#### Transition probabilities

The proportion of patients receiving PT prior to the planned invasive procedure in the PT group was assumed to be 100% based on clinical expert opinion. That in the LUSU group was calculated as 19.35% using the OR (0.04) estimated from the meta‐analysis and the pooled rate of PT group (85.71%). The proportion of patients with bleeding in the PT group was estimated as 33.33% by pooling data of L‐PLUS1 and P2b, and that in the LUSU group was calculated as 18.03% using the OR (0.44) estimated from the meta‐analysis. Proportions of patients with delay of the planned invasive procedure in both groups were based on the NICE report.^15^ Proportions of patients receiving rescue therapy following bleeding in both groups were estimated by pooling data of L‐PLUS1 and P2b.

The risk of LUSU‐related AE (i.e., severe thrombosis) was 1.56% based on pooling data of L‐PLUS1 and P2b. As a PT‐related AE, severe allergic reaction was considered in the model. The risk of severe allergic reaction was estimated as 0.05% by multiplying the incidence rate per pack of platelets (0.04%^16^) by the number of packs administered per day (1.17 [data from the Medical Data Vision Co., Ltd.—MDV—claims data analysis]). Other PT‐related AEs, such as transfusion‐related acute lung injury, infection with hepatitis A, B, C virus, human immunodeficiency virus (HIV), parvovirus B19, or prion disease, were not included in the analysis because their incidence is extremely low and hence would have a de minimis effect on the results. Bleeding‐related mortality was set as 0.83% by referring to the report of Takaki et al.^17^ The mortality rate of severe allergic reaction of PT was 10%.^18^ The annual mortality rate in each Child–Pugh category (A, B, and C) of CLD patients was estimated using the report of the median survival time by Child–Pugh category.^12^ The proportions of Child–Pugh category A, B, and C were calculated by pooling data of L‐PLUS1 and P2b. Transition probabilities used in the analysis are summarized in Table 1.

**Table 1:** Transition probabilities

Model parameter			Value	Setting in one‐way SA			Source
				Lower limit	Upper limit	Range	
Efficacy							
Proportion of patients receiving PT prior to the planned invasive procedure		PT group	100%	—	—	—	UK model (based on clinical expert opinions)^15^
		LUSU group	19.35% (OR: 0.04)	10.71% (OR: 0.02)	39.76% (OR: 0.11)	95% CI	Meta‐analysis of L‐PLUS1, P2b^13^, ^14^
Proportion of patients with bleeding		PT group	33.33%	21.09%	46.83%	±20%	Pooled data of L‐PLUS1, P2b^13^, ^14^
		LUSU group	18.03% (OR: 0.44)	8.68% (OR: 0.19)	33.55% (OR: 1.01)	95% CI	Meta‐analysis of L‐PLUS1, P2b^13^, ^14^
Proportion of patients not receiving their planned invasive procedure	Received PT prior to procedure	PT group	10.32%	6.64%	14.68%	±20%	Pooled data of L‐PLUS1, L‐PLUS2 and P2b^13^, ^14^, ^19^
		LUSU group	4.44%	2.87%	6.34%	±20%	
	No PT prior to procedure	PT group	9.09%	5.86%	12.94%	±20%	
		LUSU group	5.47%	3.53%	7.80%	±20%	
Proportion of patients receiving rescue therapy following bleeding	Received PT prior to procedure	PT group	25.00%	15.94%	35.32%	±20%	Pooled data of L‐PLUS1, P2b^13^, ^14^
		LUSU group	0%	—	—	—	
	No PT prior to procedure	PT group	0%	—	—	—	
		LUSU group	42.86%	26.82%	59.70%	±20%	
Safety							
LUSU‐related AE incidence (severe thrombosis)			1.56%	0.04%	5.69%	95% CI	Pooled data of L‐PLUS1, P2b^13^, ^14^
PT‐related AE incidence (severe allergic reaction) (% per day receiving transfusion)			0.05%	0.03%	0.07%	±20%	The Japan Society of Transfusion Medicine and Cell Therapy,^16^ claims data analysis^†^ ^‡^
Mortality							
PT‐related mortality (severe allergic reaction)			10%	6%	14%	±20%	van Eerd et al.^18^
Bleeding‐related mortality			0.83%	0.02%	3.03%	95% CI	Takaki et al.^17^
Annual mortality in CLD patients^§^		Child–Pugh A	0.099	—	—	—	Shindo et al.^12^
		Child–Pugh B	0.139	—	—	—	
		Child–Pugh C	0.462	—	—	—	
Proportion of severity in CLD patients^¶^		Child–Pugh A	52%	—	—	—	Pooled data of L‐PLUS1, P2b^13^, ^14^
		Child–Pugh B	48%	—	—	—	
		Child–Pugh C	0%	—	—	—	

^†^ 0.04% (incidence rate per pack) × 1.17 (number of packs administered per day) = 0.05% (incidence rate per day receiving transfusion).

^‡^ Calculated by multiplying the incidence rate per pack of platelet preparations (the Japan Society of Transfusion Medicine and Cell Therapy 2019) by the number of packs administered per day (claims data analysis).

^§^ Annual mortality in CLD patients = (−ln[0.5])/50% survival time × 12.

^¶^ The annual mortality in CLD patients by weighted averaging of the survival rates by severity in each analysis cycle.

AE, adverse event; CI, confidence interval; CLD, chronic liver disease; LUSU, lusutrombopag; One‐way SA, one‐way sensitivity analysis; OR, odds ratio; P2b, phase 2b trial; PT, platelet transfusion.

#### Costs and healthcare utilization

To set cost parameters, claims data from the MDV dataset were analyzed.^20^ One thousand seven hundred and eighty‐four eligible patients (LUSU, 455; PT, 1329) were used in the analysis based on the following criteria: (LUSU, 455; PT, 1329) at least one medical examination for liver disease (defined as International Classification of Diseases [ICD]‐10 code; B18, B19, C22, or K70‐77) and were prescribed platelet products (Anatomical Therapeutic Chemical [ATC] code: B02D8) or LUSU in the same month during April 2008–October 2019. Definitions of cost calculations and flow chart of patient entry are summarized in Table S2 and Figure S3. The cost distributions of each calculated item were investigated, and outlier processing was performed for cost items by excluding the value in the top 1% or greater.

Days of receiving PT were calculated from pooled data of L‐PLUS1 and P2b. The additional length of stay for delays of planned procedures was set as 4 days based on expert opinion. Costs for rescue therapy were calculated by summing the PT cost per day and PT‐related AE (severe allergic reaction, incidence rate is 0.05%) treatment cost. Cost parameters used in the analysis are summarized in Table 2.

**Table 2:** Costs

Model parameter			Value		Setting in one‐way SA			Source
					Lower limit	Upper limit	Range	
Costs of PT prior to procedure	PT cost per day	PT cost per unit	8169 JPY		—	—	—	Claims data analysis
		Units per day	12.8 units		7.8	17.9	±20%	
		Procedure fee for PT	15 380 JPY		9351 JPY	21 408 JPY	±20%	
	Days receiving PT	LUSU group	1.23		0.75	1.71	±20%	Pooled data of L‐PLUS1, P2b^13^, ^14^
		PT group	1.17		0.71	1.63	±20%	
Drug costs of LUSU		Prescribed dose	7.1 tablets		—	—	—	Claims data analysis
		Drug price (JPY/tablet)	15 586.6 JPY		—	—	—	The NHI drug price standard
Costs for delayed procedures	Additional length of stay for delays to planned procedures		4 days		—	—	—	Experts' opinion
	Hospitalization cost per day		84 451 JPY		51 347 JPY	117 555 JPY	±20%	Claims data analysis
Costs for rescue therapy following bleeding	Transfusion cost per day		120 206 JPY		—	—	—	Assumption^†^
	AE treatment cost considering incidence of complications of PT^‡^		59 JPY		—	—	—	
LUSU‐related AE treatment cost (severe thrombosis)			1 203 531 JPY		731 756 JPY	1 675 306 JPY	±20%	Claims data analysis
Costs of treating PT‐related AEs (severe allergic reaction)			120 820 JPY		73 459 JPY	168 180 JPY	±20%	Chichibu Medical Care Meeting^21^
Procedure cost	Microwave coagulation therapy		0.7%	714 372 JPY	571 498 JPY	857 246 JPY	±20%	Claims data analysis
	Paracentesis		42.3%	3 769 899 JPY	3 015 919 JPY	4 523 879 JPY	±20%	
	Hepatic artery embolization/Transcatheter arterial chemo‐embolization/Hepatic arterial chemotherapy		10.9%	913 100 JPY	730 480 JPY	1 095 720 JPY	±20%	
	Percutaneous ethanol injection therapy		0.4%	442 790 JPY	354 232 JPY	531 348 JPY	±20%	
	Radiofrequency ablation		14.8%	600 726 JPY	480 581 JPY	720 871 JPY	±20%	
	Percutaneous needle biopsy		4.7%	1 159 159 JPY	927 327 JPY	1 390 991 JPY	±20%	
	Percutaneous endoscopic gastrostomy		0.3%	4 510 316 JPY	3 608 253 JPY	5 412 379 JPY	±20%	
	Endoscopy with scheduled tissue biopsy		1.7%	4 065 726 JPY	3 252 581 JPY	4 878 871 JPY	±20%	
	Endoscopic polypectomy		2.7%	609 251 JPY	487 401 JPY	731 101 JPY	±20%	
	Endoscopic retrograde biliary drainage/Metallic stent placement		0.3%	1 421 568 JPY	1 137 254 JPY	1 705 882 JPY	±20%	
	Endoscopic variceal ligation		12.3%	1 168 041 JPY	934 433 JPY	1 401 649 JPY	±20%	
	Endoscopic injection sclerotherapy		7.9%	911 414 JPY	729 131 JPY	1 093 697 JPY	±20%	
	Endoscopic submucosal dissection/Endoscopic mucosal resection		0.1%	2 652 498 JPY	2 121 998 JPY	3 182 998 JPY	±20%	
	Dental extraction		0.1%	233 134 JPY	186 507 JPY	279 761 JPY	±20%	
	Peripherally inserted central catheter		0.9%	3 368 878 JPY	2 695 102 JPY	4 042 654 JPY	±20%	

^†^ Assuming PT for a day as rescue therapy, the cost is set by adding the transfusion cost per day to the PT‐related AE treatment cost.

^‡^ 0.05% (incidence of severe allergic reaction) × 120 820 JPY (treatment cost of treatment for severe allergic reaction) = 59 JPY (Treatment cost considering AE incidence).

AE, adverse event; LUSU, lusutrombopag; one‐way SA, one‐way sensitivity analysis; P2b, phase 2b trial; PT, platelet transfusion.

#### Utility

Utility values set in the model were collected by literature review. The baseline utility of CLD patients was set as 0.808 from the mean value of chronic hepatitis (0.871), liver cirrhosis (0.774), and hepatocellular carcinoma (HCC) (0.78) reported by Kaishima et al.^22^ Disutility values of severe thrombosis, severe allergic reaction, and bleeding were set as −0.029,^23^ −0.4,^18^ and −0.397,^23^ respectively, and the influence of these events on quality of life (QOL) was assumed to last for 1, 4, and 1 week, respectively. No disutility for delays to planned procedures was considered. In the Markov phase, the utility values of survival were adjusted to address the impact of aging using the regression equation adopted from each sex and age group in the general Japanese population reported by Shiroiwa et al.^24^ Utility values used in the analysis are summarized in Table 3.

**Table 3:** Utilities

Model parameter	Value	Setting in one‐way SA			Source
		Lower limit	Upper limit	Range	
Baseline in CLD patients	0.808	0.44	0.99	±20%	Kaishima et al.^22^
LUSU‐related AE (severe thrombosis)	−0.029	−0.02	−0.04	±20%	Jugrin et al.^23^
PT‐related AE (severe allergic reaction)	−0.4	−0.25	−0.56	±20%	van Eerd et al.^18^
Delays to planned invasive procedures	0	—	—	—	Assumption
Bleeding	−0.397	−0.55	−0.25	±20%	Jugrin et al.^23^
General population	Regression equation^†^: Utility value in general population = 0.9978786 + 0.0218333 × Male% − 0.002153 × Age				Shiroiwa et al.^24^

^†^ Age: Age at each cycle, Male%: Male% at each cycle (estimated male‐female ratio in the analysis population at each cycle by applying mortality rate by sex of the general population to the male–female ratio at baseline).

AE, adverse event; CLD, chronic liver disease; LUSU, lusutrombopag; one‐way SA, one‐way sensitivity analysis; PT, platelet transfusion.

### Model outcomes

The cost‐effectiveness of LUSU was evaluated using the incremental cost‐effectiveness ratio (ICER) calculated by dividing the incremental cost relative to the PT by the incremental QALY. The threshold of ICER in this analysis was set at 5 million JPY/QALY.^25^, ^26^ Cases in which the ICER was lower than 5 million JPY/QALY were rated as cost‐effective.

The initial age was set as 68 years old based on the mean age of the patients in L‐PLUS1 and P2b, assuming all patients in the PT group received PT prior to the procedure. In addition to the base‐case analysis, three scenario analyses were conducted. In scenario A, in contrast to the base‐case, the number of days for pre‐procedure PT for both groups to be 1 day, reflecting clinical expert opinion. In scenario B, the proportion of patients receiving PT prior to the procedure in the PT group was set as 85.71% based on pooled data of L‐PLUS1 and P2b. In scenario C, the time horizon of the analysis was set as 35 days (the study period as defined within the LUSU clinical trials).

### Sensitivity analysis

The one‐way sensitivity analysis (SA) was conducted for the base‐case analysis to explore uncertainty in parameters that may affect the ICER. The one‐way SA range for ORs and the incidence of severe thrombosis was set to the 95% CI for each value. For all parameters without a reported or estimated 95% CI, the range of ±20% was used. The top 10 variables in the one‐way SA are shown in a tornado diagram regarding net monetary benefit.

The robustness of the base‐case result was examined by probabilistic sensitivity analysis (PSA), which simultaneously changes multiple parameters using Monte Carlo simulation with 1000 iterations. The PSA was assumed to have a lognormal distribution for ORs, and a beta distribution for transition probabilities and utility values, whereas a normal or partially beta distribution was applied for the number of units of PT and costs. The distributions were set using the standard error (SE) of each parameter. For some parameters, 20% of the setting value was defined as SE if it could not be estimated or was not available in the literature.

## Results

### Base‐case analysis and scenario analysis

The results of the base‐case analysis and scenario analysis are summarized in Table 4. In the base‐case, LUSU and PT gained 6.1803 QALYs and 6.1712 QALYs, and the expected lifetime costs were 2 380 219 JPY and 2 382 908 JPY, respectively. Thus, LUSU was evaluated as dominant (cost‐saving) compared with PT. In scenarios A and B, LUSU was shown to be cost‐effective *versus* PT at 1352691 JPY/QALY in scenario A and 2 135 211 JPY/QALY in scenario B. In scenario C as in the base‐case, LUSU was dominant (cost‐saving) *versus* PT.

**Table 4:** Analysis results

Strategy	Cost (JPY)	Incremental cost (JPY)	QALY	Incremental QALY	ICER (cost/QALY)
Base‐case					
LUSU	2 380 219	−2689	6.1803	0.0091	Dominant
PT	2 382 908	—	6.1712	—	—
Scenario A: Assuming the number of days receiving PT prior to procedure as 1 day in both groups					
LUSU	2 374 847	12 362	6.1803	0.0091	1 352 691 JPY
PT	2 362 485	—	6.1712	—	—
Scenario B: Using pooled data of Japanese clinical trials (L‐PLUS1, P2b) as proportion of patients receiving PT in the PT group					
LUSU	2 380 219	19 417	6.1803	0.0091	2 135 211 JPY
PT	2 360 801	—	6.1713	—	—
Scenario C: Time horizon of 35 days					
LUSU	2 380 219	−2689	0.0760	0.0012	Dominant
PT	2 382 908	—	0.0749	—	—

Scenario A: The number of days for pre‐procedure PT for both groups to be 1 day (base‐case, LUSU group **=** 1.23 days; PT group **=** 1.17 days). Scenario B: The proportion of patients receiving PT prior to the procedure in the PT group was set as 85.71% (base‐case, PT group **=** 100%). Scenario C: The time horizon of the analysis was set as 35 days (base‐case, 50 years [a lifetime]).

ICER, incremental cost‐effectiveness ratio; LUSU, lusutrombopag; P2b, phase 2b trial; PT; platelet transfusion; QALY, quality‐adjusted life year.

### Sensitivity analysis

**Figure 2:**
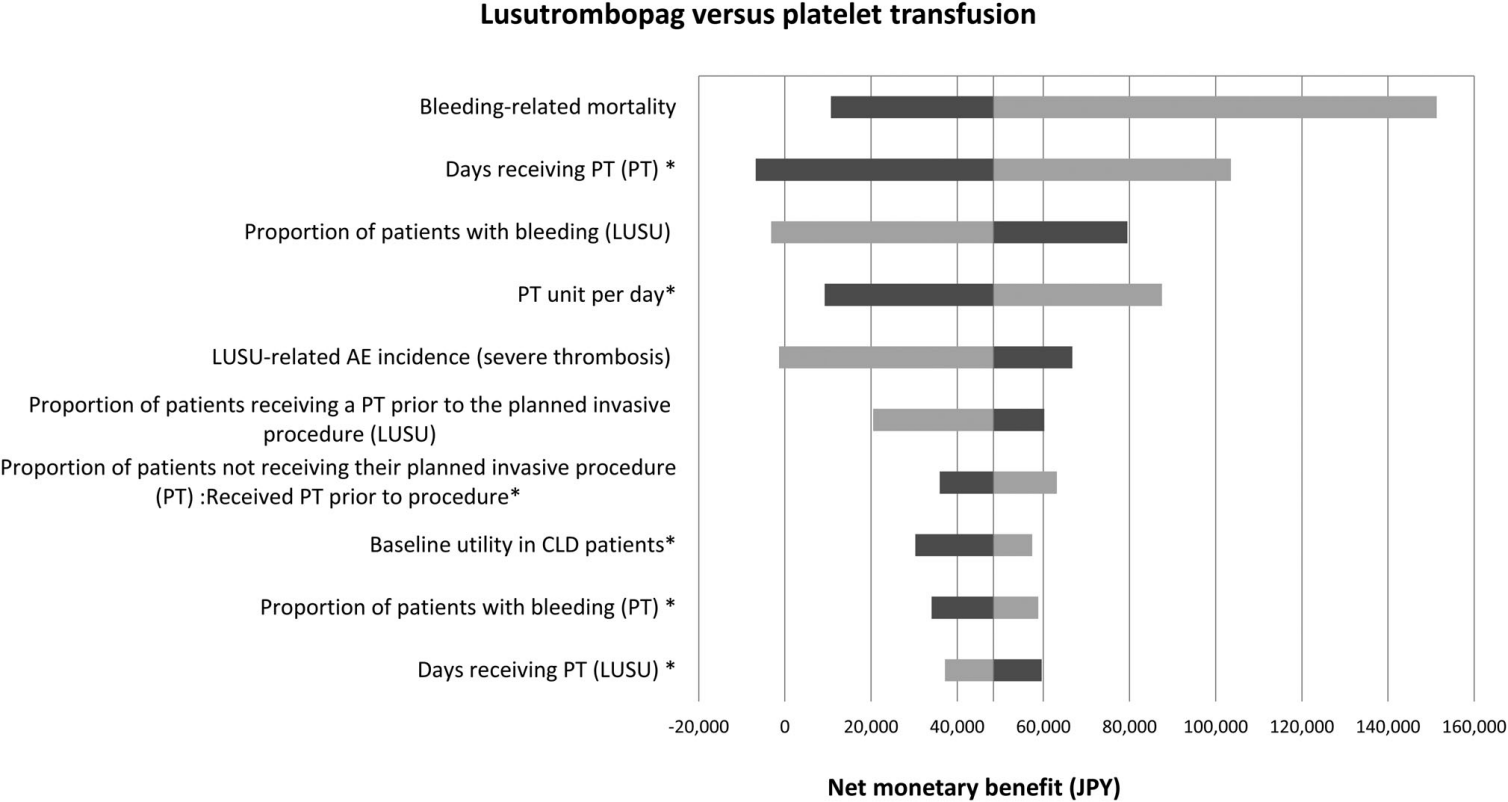
Result of one‐way sensitivity analysis using the tornado diagram.CI, confidence interval; OR, odds ratio; PT, platelet transfusion. *Parameters with range ± 20% not 95%CI. 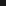, Lower bound; 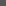, upper bound.

The results of one‐way SA using the tornado diagram are shown in Figure 2. The top three parameters affecting the analysis results were “bleeding‐related mortality,” “number of PT prior to surgery in the PT group,” and “risk of patients with bleeding in the LUSU group.”

**Figure 3:**
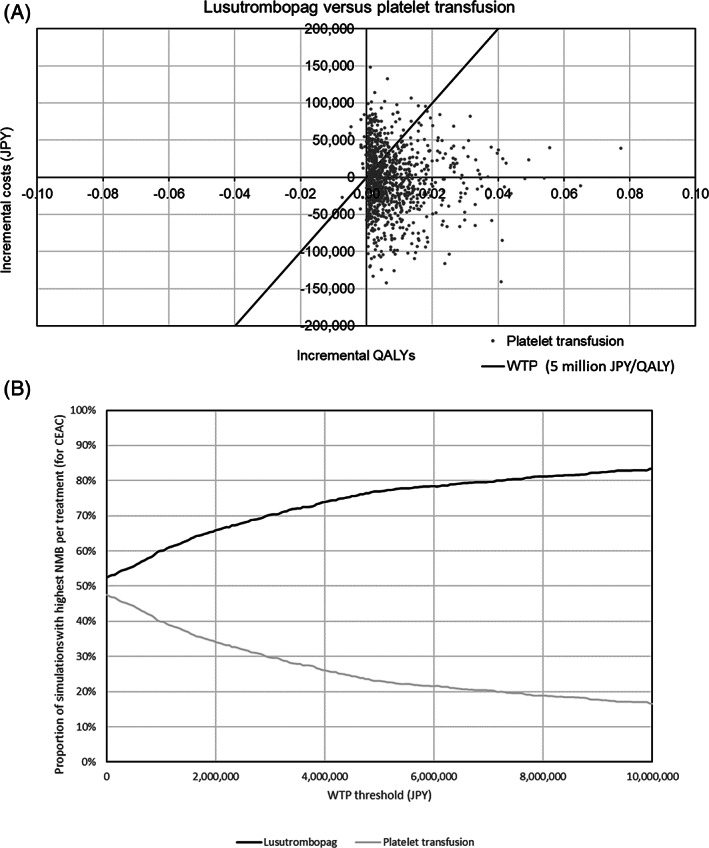
Result of probabilistic sensitivity analysis.(A) Cost‐effectiveness plane. (B) Cost‐effectiveness acceptability curve. CEAC, cost‐effectiveness acceptability curve; QALY, quality‐adjusted life year; WTP, willingness to pay.

In the PSA, the probability of LUSU being dominant was 51.8%, and the ICER was less than 5 million JPY/QALY in 78.3% of simulations (Fig. 3).

## Discussion

In this analysis, the cost‐effectiveness of LUSU compared with PT in Japan was evaluated using a cost‐effectiveness model relative to the Japanese healthcare system. As a result, LUSU treatment for CLD patients with thrombocytopenia (platelet count <50 × 10^9^/L) who required a planned invasive procedure was evaluated as dominant (cost‐saving) to PT in Japan. Clinical evidence of LUSU evaluated in Japanese, multicenter, randomized, double‐blind, parallel‐group, placebo‐controlled studies was used in the analysis. Most of the cost parameters were calculated using a Japanese claims database to reflect real‐world data in Japan.

The probability of LUSU being dominant (cost‐saving) and of the ICER being below 5 million JPY/QALY were estimated at 51.8% and 78.3%, respectively, demonstrating the robustness of the results. Moreover, in scenario C, in which the time horizon of the analysis was set as the period undergoing an invasive procedure (35 days), LUSU was dominant, as in the base‐case analysis. One course of LUSU is expected to prevent AEs and complications by reducing the use of PT and days of hospitalization. However, as our model did not account for these variables, the results of our analysis are conservative and do not include these additional costs. Recently, Armstrong et al. reported a systematic review and CEA of thrombopoietin receptor agonists avatrombopag ((Doptelet®; Dova Pharmaceuticals, Durham, NC, USA) and LUSU for thrombocytopenia in people with CLD needing an elective procedure.^27^ Although it is not appropriate to compare our result with them due to differences in the medical environment and the model structure used in the analysis, they concluded that avatrombopag and LUSU were superior to no thrombopoietin receptor agonist in avoiding both PT and rescue therapy, but they were not cost‐effective given the lack of benefit and increase in cost under the UK setting. As part of the decision‐making, NICE analyzed the Armstrong findings and deemed that there were several lack of data considerations that flaw this conclusion. The NICE Committee did agree with Shionogi in relation to the economic evaluation results of LUSU.

To our knowledge, this is the first study to evaluate cost‐effectiveness of treating thrombocytopenia prior to a planned invasive procedure in patients with CLD in Japan. This study revealed that LUSU can be a cost‐saving and efficient treatment option for Japanese patients with CLD whose thrombocytopenia needs to be treated prior to a planned invasive procedure.

There are several limitations to address in terms of the interpretation of the results. First, as there was only one event in both groups regarding the proportion of patients not receiving their planned invasive procedure, a reliable evaluation was impossible by meta‐analysis of two trials (L‐PLUS1 and P2b). These data were also unable to be obtained from the MDV claims database; thus whether the invasive procedure was delayed was unknown. Although this proportion should be evaluated using a database linked to medical record information or launch of a registry study, the one‐way SA demonstrated that the impact of the proportion on the CEA result was small. Second, regarding the number of days for pre‐procedure PT, the number of events was insufficient to perform comparative evaluation by meta‐analysis. Based on one‐way SA, the value greatly impacted the CEA results. However, even in the scenario A analysis assuming the number of days for pre‐procedure PT for both groups to be 1 day, the ICER was well below 5 million JPY/QALY and LUSU remained cost‐effective *versus* PT. Lastly, disutility values due to AE were referred to EU data because no Japanese data were available. It might not be representative of results based on Japanese population. However, the one‐way SA demonstrated the impact of these disutility values on the CEA result to be small.

In conclusion, although the analysis has several limitations, it substantiates that LUSU is cost‐savings to the Japanese health system under certain assumptions (base‐case) and cost‐effective when critical parameters are analyzed through a one‐way SA. Hence, LUSU represents good value for money as an efficient treatment option for thrombocytopenia in CLD patients prior to a planned invasive procedure.

In this study, LUSU was evaluated as a valuable treatment option for Japanese patients with CLD patients who required treatment of their thrombocytopenia prior to a planned invasive procedure.

## Supporting information


**Table S1.** Patient characteristics in L‐PLUS1 and P2b.
**Table S2.** List of claims data analysis.
**Figure S1.** Transfusion requirements; a platelet transfusion prior to the planned invasive procedure.
**Figure S2.** Bleeding following a planned or rescheduled invasive procedure.
**Figure S3.** Flow chart of patient entry.
