# Supplementary material for: A cost‐effectiveness analysis of lusutrombopag for thrombocytopenia in patients with chronic liver disease in Japan
Source: JGH Open. 2021 Jun 26;5(8):879–87. doi: 10.1002/jgh3.12597 (PMC8341178; doi:10.1002/jgh3.12597)
Supplement: Supplementary file 1 — Table S1. Patient characteristics in L‐PLUS1 and P2b. Table S2. List of claims data analysis. Figure S1. Transfusion requirements; a platelet transfusion prior to the planned invasive procedure. Figure S2. Bleeding following a planned or rescheduled invasive procedure. Figure S3. Flow chart of patient entry. [file JGH3-5-879-s001.docx]

**Supporting Information**

Table S1. Patient characteristics in L-PLUS1 and P2b.

| Item | L-PLUS1 | | P2b | |
| --- | --- | --- | --- | --- |
|  | **LUSU 3 mg**  **n=48** | **Placebo**  **n=48** | **LUSU 3 mg**  **n=16** | **Placebo**  **n=15** |
| Age (mean ± SD) | 68.9 (6.6) | 66.8 (10.2) | 66.8 (8.1) | 70.9 (8.6) |
| Male, n (%) | 21 (43.8) | 30 (62.5) | 9 (56.3) | 8 (53.3) |
| Child-Pugh class  A, n (%)  B, n (%) | 26 (54.2)  22 (45.8) | 22 (45.8)  26 (54.2) | 9 (56.3)  7 (43.8) | 9 (60.0)  6 (40.0) |
| Proportion of patients receiving PT prior to the planned invasive procedure | 10/48 | 42/48 | 3/16 | 12/15 |
| Proportion of patients not receiving their planned invasive procedure | 0/48 | 1/48 | 1/16 | 0/15 |
| Proportion of patients with bleeding | 7/48 | 13/48 | 5/16 | 8/15 |
| Proportion of patients receiving rescue therapy following bleeding | 0/48 | 1/48 | 1/16 | 0/15 |

LUSU, lusutrombopag; P2b, phase 2b trial; PT, platelet transfusion; SD, standard deviation.

Table S2. List of claims data analysis

| **Calculated Item** | **Definition** |
| --- | --- |
| (1) Calculation of prescribed dose of LUSU | |
| 1) Prescribed dose of LUSU | Number of Mulpleta tablets 3 mg prescribed |
| (2) Calculation of LUSU-related AE treatment cost | |
| 1) Severe thrombosis | Hospitalization cost per hospitalization due to portal vein thrombosis |
| (3) Calculation of PT costs prior to procedure | |
| 1) Days receiving PT (by treatment group) | Number of days receiving PT prior to an invasive procedure |
| 2) Units of transfusion per day | Number of units of transfusion per day receiving PT |
| 3) Procedure fee for PT | Medical fee of blood transfusion per day |
| 4) Proportion of platelet preparations | Administration rate of each platelet preparation |
| 5) Proportion of day case PT | (3)- 1) Proportion of days receiving outpatient PT among days receiving PT |
| 6) Proportion of inpatient PT | (3)- 1) Proportion of days receiving inpatient PT among days receiving PT |
| 7) Cost for day case PT | Total cost combined with platelet preparation cost per day for outpatient PT and procedure fees during the evaluation period |
| 8) Cost for inpatient PT | Total cost combined with platelet preparation cost per day for inpatient PT and procedure fees during the evaluation period |
| (4) Calculation of invasive procedure costs^†^ | |
| 1) Proportion of each invasive procedure | Breakdown of each invasive procedure in target population |
| 2) Costs of each invasive procedure | A series of outpatient or inpatient treatment costs for invasive procedures |
| (5) Calculation of hospitalization cost per day for setting costs for a delayed procedure | |
| 1) Hospitalization cost per hospitalization | Hospitalization cost per hospitalization receiving each invasive procedure |
| 2) Length of stay | Length of stay per hospitalization receiving each invasive procedure |
| 3) Hospitalization cost per day | Hospitalization cost per day for hospitalization receiving each invasive procedure |

AE, adverse event; LUSU, lusutrombopag; PT, platelet transfusion.

† A part of types of invasive procedures included are changed from the global model based on the opinions of Japanese clinicians.

Figure S1. Transfusion requirements; a platelet transfusion prior to the planned invasive procedure.


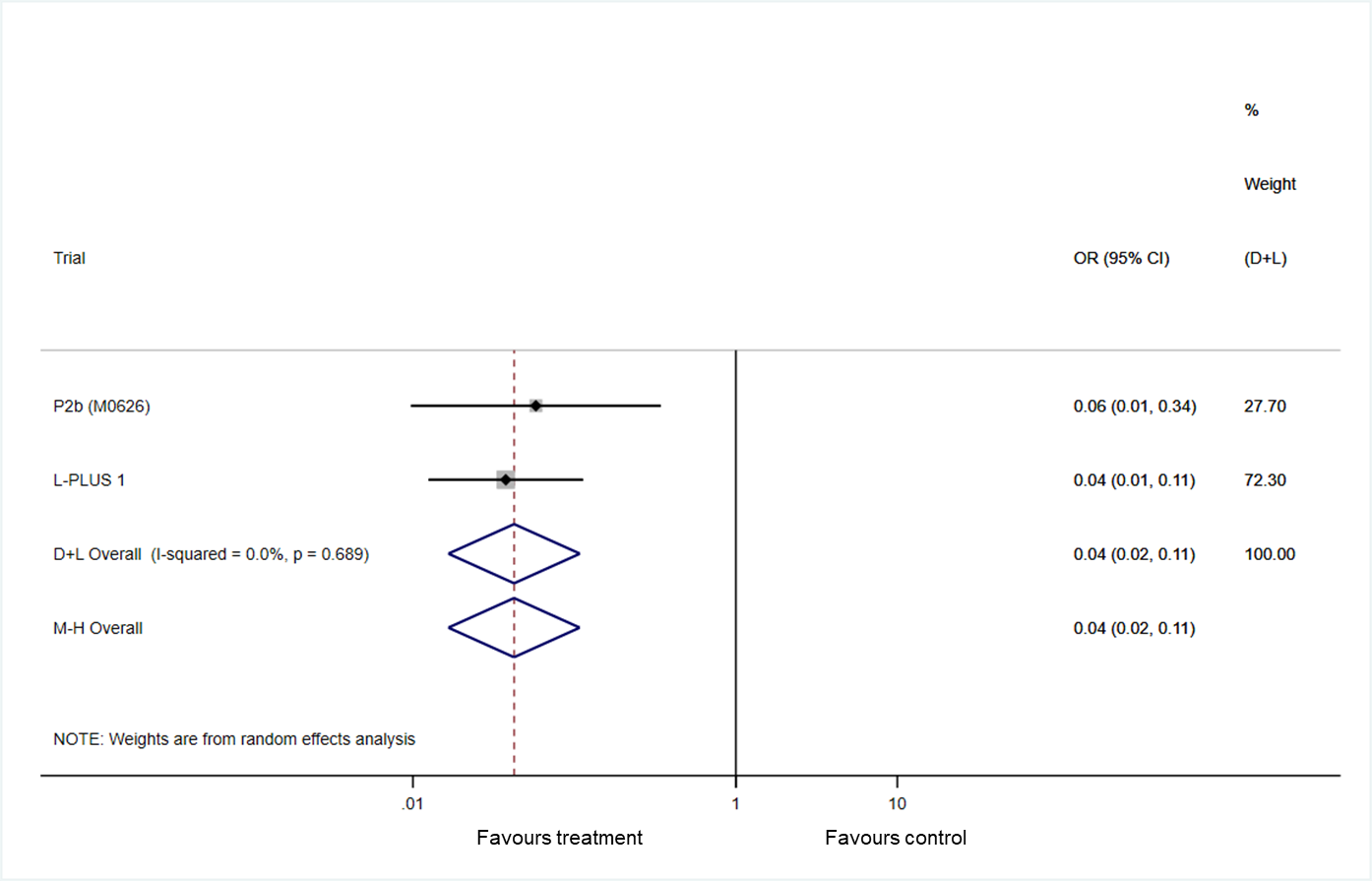


CI, confidence interval; OR, odds ratio; P2b, phase 2b trial.

Figure S2. Bleeding following a planned or rescheduled invasive procedure.


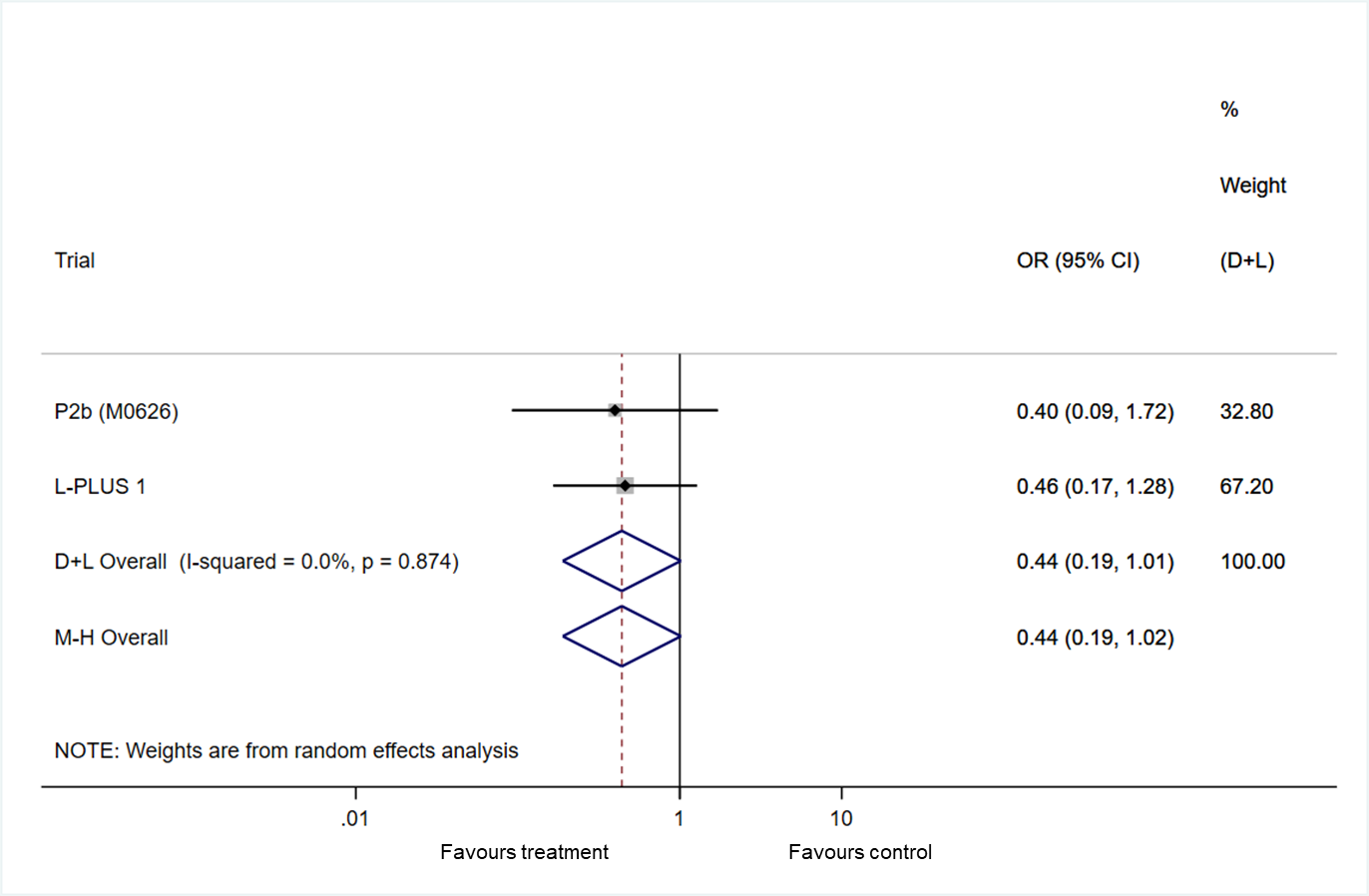


CI, confidence interval; OR, odds ratio; P2b, phase 2b trial.

Figure S3. Flow chart of patient entry.


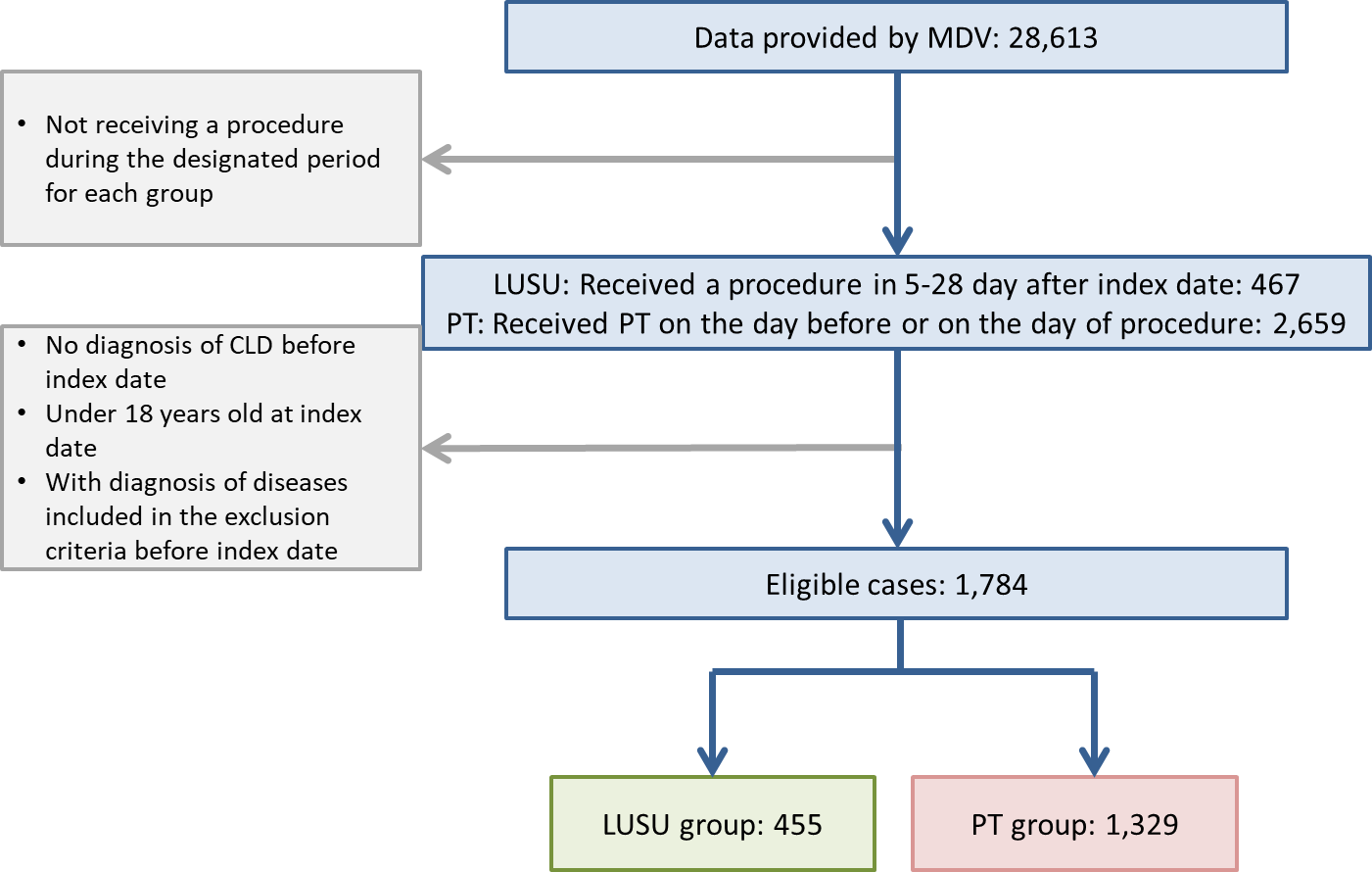


CLD, chronic liver disease; LUSU, lusutrombopag; MDV, Medical Data Vision Co., Ltd; PT, platelet transfusion.
